# Health literacy and diabetic retinopathy

**DOI:** 10.1590/1414-431X2023e13066

**Published:** 2024-01-22

**Authors:** J.C. Breder, I. Breder, J. Barreto, V. Fernandes, F. Zanchetta, B.A. Oliveira, F. Chaves, A. Sposito, M.H.M. Lima

**Affiliations:** 1 Faculdade de Enfermagem, Universidade Estadual de Campinas, Campinas, SP, Brasil; 2 Departamento de Cardiologia, Laboratório de Aterosclerose e Biologia Vascular, Universidade Estadual de Campinas, Campinas, SP, Brasil; 3 Departamento de Oftalmologia, Hospital de Clínicas, Universidade Estadual de Campinas, Campinas, SP, Brasil; 4 Faculdade de Ciências Médicas, Universidade Estadual de Campinas, Campinas, SP, Brasil

**Keywords:** Health literacy, Diabetic retinopathy, Diabetes Mellitus

## Abstract

Health literacy (HL) is defined as a cognitive and social skill that determines the motivation and ability of individuals to understand and use information to promote and maintain proper health. Inadequate HL has been associated with worse outcomes in diabetes control, poor self-care, and higher hospitalization rates for some chronic diseases. We hypothesized that HL influences the prevalence of diabetic retinopathy (DR) among individuals with type 2 diabetes mellitus (T2DM) and that inadequate glycemic control would mediate this association. This was a cross-sectional study carried out with 288 participants of the “Brazilian Diabetes Study” cohort. Inclusion criteria were people diagnosed with T2DM aged between 40 and 70 years and ability to read and write. In the adequate HL group, DR was found in 16.5% of participants and in the inadequate HL group, it was found in 32.8% (P=0.0081). Individuals with inadequate HL had a higher risk of having DR, and this association was still statistically significant after adjusting for HbA1c, low-density lipoprotein cholesterol, systolic blood pressure, and diastolic blood pressure. In conclusion, HL is related to DR without the mediation of classical clinical variables.

## Introduction

The World Health Organization defines health literacy (HL) as the cognitive and social skill of individuals to access, understand, and use information in a way that promotes and maintains adequate health (1).

In people with type 2 diabetes mellitus (T2DM), HL is related to the understanding of self-care and disease management, which is intrinsically linked to clinical outcomes (2).

Low HL has been associated with worse outcomes in diabetes control, poor self-care, higher hospitalization rates (2,3), lower use of preventive services (3), delay in diagnosis (4), less knowledge about health (2,3), and increased risk of mortality (3). On the other hand, patients with adequate HL reported better adherence to diet, blood glucose monitoring, and foot care (5).

The International Diabetes Federation (IDF) estimates that 537 million adults aged between 20 and 79 live with diabetes. This represents 10.5% of the world's population in this age group. This number is expected to increase to 643 million (11.3%) by 2030 and to 783 million (12.2%) by 2045 (6). Diabetic retinopathy (DR) is one of the most common and serious microvascular complications in diabetes (7,8).

DR affects 1 in 5 people with T2DM and is the leading and still growing cause of blindness worldwide, particularly in low-to-middle-income countries (7). DR is one of the tissue injuries resulting from T2DM most intensely related to glycemic control (9). Based on these premises, we hypothesized that HL influences the prevalence of DR in individuals with T2DM and that inadequate glycemic control could link this association. The present study was designed to investigate these hypotheses.

## Material and Methods

This was a cross-sectional study carried out with 288 participants of the “Brazilian Diabetes Study” (BDS) cohort, which is a prospective, ongoing, single center, cohort of T2DM (clinicaltrials.gov: NCT04949152). Clinical and laboratory analyses were performed by the Atherosclerosis and Vascular Biology Laboratory (Aterolab) at the Clinical Research Center of the State University of Campinas (Unicamp), Brazil. The study was approved by the Research Ethics Committee of Unicamp (CAAE: 89525518.8.1001.5404) and complied with the principles of the Declaration of Helsinki (10). To calculate the sample size of the study, we considered all participants in the BDS cohort who had undergone the ophthalmological evaluation, and the number obtained was 308 participants. A sampling error of 3% and a significance level of 5% were assumed. Thus, the minimum sample size required was 239 patients.

Eligible patients (blood sample collection, ambulatory blood pressure monitoring, ophthalmologic evaluation) were invited to the research center for an explanation of the study protocol. Inclusion criteria were people diagnosed with T2DM, aged between 40 and 70 years, ability to read and write. After signing the written informed consent, demographic data were collected from all participants and blood pressure was measured. Blood samples were also collected after 12-h fasting.

Blood pressure was measured using the HEM-7113 Omron Healthcare device (Brazil), as stated in the latest guideline (11). After 12-h fasting, peripheral blood was collected following the appropriate instructions (12). DR staging was performed by a retinal ophthalmology specialist. After clinical evaluation, patients were submitted to complementary examinations of retinography in a VISUCAM device (NM/FA Carl Zeiss, Germany) and optical coherence tomography (OCT) using SPECTRALIS SD-OCT (Heidelberg Engineering GmbH, Germany). DR was classified as not apparent or present (minimal non-proliferative diabetic retinopathy, mild to moderate, severe, very severe, and early or high-risk proliferative diabetic retinopathy) (8).

To assess HL, the SAHLPA-18 (Short Assessment of Health Literacy for Portuguese-speaking Adults) instrument was used, with one point for each correct item with a maximum score of 18. We categorized patients as having inadequate functional HL if the SAHLPA-18 score was 0-14 and adequate functional HL if it was 15-18. (13). The interview was conducted by the researchers after adequate training on the specific test in a room exclusively reserved for this purpose.

The clinical research data management was based on the Research Electronic Data Capture (REDCap, USA) platform. Continuous variables are reported as median and interquartile range (IQR). We used the Mann-Whitney test to compare continuous variables and the chi-squared test for categorical variables. Modified Poisson regression models with robust variance were used for the analysis of the association between HL and DR. Covariates in this modeling were high glycated hemoglobin [(HbA1c) >7% (53 mmol/mol)], high low-density lipoprotein cholesterol [(LDL-C); >50 mg/dL in secondary prevention or >70 mg/dL in primary prevention], high systolic blood pressure [(SBP) >130 mmHg], and high diastolic blood pressure [(DBP) >90 mmHg] (14). Simple mediation analyses were performed to investigate if the association between HL and DR was mediated by glycemic control. In this model, the predictive variable was HL, the mediator was HbA1c, and the outcome variable was DR; the Sobel test was used. HbA1c, LDL-C, SBP, and DBP were entered as mediating variables (15). A P-value <0.05 was considered statistically significant. SAS software version 9.4 (USA) was used to perform statistical analysis.

## Results

Table 1 presents the demographics of the study participants classified as adequate or inadequate HL groups. While in the adequate HL group, DR was found in 16.5% of participants, in the inadequate HL group, it was found in 32.8% (P=0.0081). No statistical difference was found between adequate HL and inadequate HL groups and severe DR: 1.76 and 1.72% (P=0.0543), respectively.

**Table 1:** Comparison of clinical characteristics of participants in literacy groups.

	Adequate HL (N=204)	Inadequate HL (N=83)	P-value
Age, years	59 [12.5]	59 [11]	0.8858*
Gender, male, %	65.67	34.33	0.0020**
Family income, BR$/month	4.000 [3.500]	3.000 [2.500]	0.0007*
Schooling, years	13 [4]	9 [7]	<0.0001*
T2DM duration, years	10 [10]	9 [11]	0.3417*
HbA1c, %	7.3 (56 mmol/mol) [1.65]	7.2 (55 mmol/mol) [2.2]	0.9084*
LDL-C, mg/dL	91 [38]	89 [51]	0.9177*
HDL-C, mg/dL	42 [15]	40 [11]	0.0678*
Office systolic blood pressure, mmHg	136 [22]	139 [20]	0.2812*
Office diastolic blood pressure, mmHg	85 [15]	84.5 [13]	0.5234

Data are reported as median and interquartile range [IQR], except gender. HL: Health literacy; BR$: Brazilian reais; HbA1c: glycated hemoglobin; LDL-C: low-density lipoprotein cholesterol; HDL-C: high density lipoprotein cholesterol; *P-value obtained with the Mann-Whitney test; **P-value obtained with the chi-squared test. P-values in bold indicate statistically significant. One participant refused to continue in the study.

As shown in Table 2, individuals with inadequate HL had a higher risk of having DR; the association remained significant after being adjusted for HbA1c, LDL-C, SBP, and DBP. As expected, high HbA1c was also related to an increased risk of DR. According to the mediation analysis, the association between DR and HL was not modulated by HbA1c [2.22%; Sobel test Z=0.142 (0.068), P=0.886]. LDL-C, SBP, and DBP were also not identified as mediating variables for this association.

**Table 2:** Multivariate analysis of the relationship between health literacy and diabetic retinopathy adjusted for clinically relevant covariates.

Dependent variable	Independent variable	Prevalence ratio*	P-value
Diabetic retinopathy	HL class. (ref=Adequate)	2.04 (1.22-3.42)	**0.0069**
Diabetic retinopathy	HL class. (ref=Adequate)	2.07 (1.25-3.42)	**0.0049**
	HbA1c >7% (53 mmol/mol)	1.92 (1.04-3.54)	**0.0373**
	High LDL-C	0.94 (0.49-1.79)	0.8400
	High SBP or DBP	0.76 (0.45-1.28)	0.3005
Diabetic retinopathy	HL class. (ref=Adequate)	2.05 (1.23-3.42)	**0.0058**
	HbA1c >7% (53 mmol/mol)	1.95 (1.05-3.60)	**0.0340**
Diabetic retinopathy	HL class. (ref=Adequate)	2.03 (1.21-3.41)	**0.0076**
	High LDL-C	0.92 (0.50-1.70)	0.7967
Diabetic retinopathy	HL class. (ref=Adequate)	2.06 (1.23-3.45)	**0.0058**
	High SBP or DBP	0.73 (0.43-1.23)	0.2391

*The probability of having some degree of diabetic retinopathy was estimated. HL class.: health literacy classification; LDL-C: low-density lipoprotein cholesterol; SBP: systolic blood pressure; DBP: diastolic blood pressure; HbA1c: glycosylated hemoglobin; class: classification. P-values in bold indicate statistically significant.

## Discussion

Consistent with previous reports, we found inadequate HL in 29% of patients with T2DM (16), and these individuals had a higher prevalence of DR (17). Our study took this investigation one step further by performing mediation analyses based on well-established risk factors for the development of DR, i.e., high levels of HbA1c, LDL-C, and BP (9). Surprisingly, we found that HbA1c had a minimal (2.22%), statistically insignificant, mediating effect in the association between HL and DR, and the same was true for traditional risk factors such as LDL-C and BP. This result opened space for discussion about the extent of relevance of HL in individuals with T2DM, extrapolating the role usually attributed to control of traditional risk factors.

On the other hand, a systematic review that investigated clinical predictors of DR progression concluded that hyperglycemia, dyslipidemia, and microalbuminuria contribute to the evolution of this complication. Progression tends to be faster when diabetes presents a long duration and the patient has low hemoglobin levels (18). Known variables such as glycemic variability and others not yet known but potentially influenced by physician-patient communication need to be clarified in future studies.

At present, our findings highlighted the importance of identifying HL in T2DM patients as a marker for the manifestation of microvascular lesions such as DR and encourage a tailored approach for these individuals (2,19). However, epidemiological projections suggest a growing increase of DR in resource-poor environments. For this reason, advances in DR management should be accessible to these populations to further reduce vision loss and blindness in DR over the next decade (20).

Our findings, however, should be interpreted with caution. Participants were not selected randomly, but were volunteers among participants who had ophthalmological evaluations, which may lead to biased results.

In conclusion, HL was associated with DR, but this effect was not mediated by the classical clinical variables.
